# Relationships between Hearing Loss and the Prevalences of Cataract, Glaucoma, Diabetic Retinopathy, and Age-Related Macular Degeneration in Korea

**DOI:** 10.3390/jcm8071078

**Published:** 2019-07-22

**Authors:** Joon Mo Kim, Se Young Kim, Hee Seung Chin, Hyun Ji Kim, Na Rae Kim

**Affiliations:** 1 Department of Ophthalmology, Kangbuk Samsung Hospital, Sungkyunkwan University School of Medicine, Seoul 03181, Korea; 2 Department of Ophthalmology and Inha Vision Science Laboratory, Inha University School of Medicine, Incheon 22332, Korea; 3 Department of Otorhinolaryngology, Inha University School of Medicine, Incheon 22332, Korea

**Keywords:** age-related macular degeneration, cataract, diabetic retinopathy, glaucoma, hearing loss

## Abstract

This study was conducted using the database of the Korea National Health and Nutrition Examination Survey to determine whether age-related eye diseases such as cataract, glaucoma, diabetic retinopathy (DR), and age-related macular degeneration (AMD), are related to hearing loss. 12,899 participants ≥ 40 years of age were included. The weighted prevalence of diabetic retinopathy was not significantly different between the normal hearing group and hearing-impaired group, but the weighted prevalences of cataract, glaucoma, early AMD, and late AMD were significantly different in the two groups. The odds ratio for cataract in the hearing-impaired group was 1.373 (1.118–1.687). The odds ratios of glaucoma, DR, early AMD, and late AMD were not significantly different in the hearing-impaired group. Age was significantly associated with the presence of concurrent cataract and hearing impairment by 6.574-fold per decade. Significant factors that increased the risk of concurrent glaucoma and hearing impairment were age, male gender, and triglyceride. Age, ex-smoker, systolic BP elevation, BMI decline, and fasting blood sugar significantly predicted the presence of concurrent DR and hearing loss. In early AMD, age and triglyceride, and in late AMD, age and systolic BP elevations increased the risk of concurrent AMD and hearing impairment.

## 1. Introduction

As people live longer, the incidences of hearing loss, vision loss, and the loss of both senses are increasing, and loss of both senses markedly diminishes quality of life. A previous study showed clear relations between all types of sensory loss and symptoms of depression and the likelihood of experiencing symptoms of depression is much higher, especially due to loss of two senses [1,2]. Dual impairment also reduces physical activity [3] and cognitive ability [4], and increases social isolation [5], driving difficulties and the risk of motor vehicle accidents [6].

The Korea National Health and Nutrition Examination Survey (KNHANES) is a population-based, cross-sectional epidemiological survey conducted by the Korean Ministry of Health and Welfare. In this survey, vision impairment is defined as a best corrected visual acuity (BCVA) of ≤0.32 in the better eye. KNHANES 2008–2009 showed the overall prevalence of visual impairment was 0.4% and that in individuals aged over 40 years it was 0.9% [7]. In Korean adults aged over 40 years of age, the prevalences of cataract, primary open angle glaucoma (POAG), diabetic retinopathy (DR), early age-related macular degeneration (AMD), and late AMD were high, at 40.2%, 2.1%, 13.4%, 5.1%, and 0.5%, respectively [7]. Furthermore, audiological testing and otologic examinations revealed prevalences of bilateral and unilateral hearing loss at speech-relevant frequencies of 9.31% and 13.42%, respectively [8]. 

Although various reports have documented eye diseases and hearing loss, no large-scale study has investigated their combined prevalence or the risk factors of specific eye diseases associated with hearing loss. In this study, we aimed to document the prevalences of eye diseases and of hearing impairment in individuals included in the KNHANES V, and to identify factors associated with concurrent eye disease and hearing impairment.

## 2. Methods

### 2.1. Participants

KNHANES was conducted by the Korea Centers for Disease Control and Prevention to assess the health and nutritional status of the Korean population. Participants were selected by georegionally-based multistage probability sampling, stratified by sex and age. Using a weighting scheme, the survey produces estimated health statistics representing the noninstitutionalized Korean population. A more detailed description of KNHANES participants has been previously reported [7]. As a part of KNHANES V, an ophthalmologic survey was conducted over a 5-year period from 2008 to 2013. Data for the present study were obtained during a cross-sectional series of interviews and examinations conducted between 2010 and 2012. The present study was approved by the institutional review board of the Korea Centers for Disease Control and Prevention (approval numbers 2010-02CON-21-C, 2011-02CON-06-C, and 2012-01EXP-01-2C), and all participants provided written informed consent.

### 2.2. Ocular Examinations

Participants in KNHANES V over 19 years of age underwent full ocular examinations, including visual acuity, refraction, and intraocular pressure (IOP) measurements, and slit-lamp and fundus examinations with photographs. Refractive errors were evaluated by autorefraction (KR-8800; Topcon Corporation, Tokyo, Japan). Bilateral IOP was measured once in the morning between 8:00 AM and 12:00 noon with Goldmann applanation tonometry and anterior segment was evaluated by slit-lamp (Haag-Streit Inc, Bern, Switzerland) by ophthalmologists. The presence of pseudophakia or aphakia was determined. Anterior chamber angle width was estimated using Van Herick’s method. Eyes with a peripheral anterior chamber depth of less than a quarter of peripheral corneal thickness were classified as narrow angle eyes. Digital fundus images were acquired using a digital non-mydriatic retinal camera (TRC-NW6S; Topcon Corporation) and a Nikon D-80 digital camera (Nikon Inc, Tokyo, Japan). Images were acquired using physiologic mydriasis. Vertical and horizontal cup-to-disc ratios (VCDRs) were measured by glaucoma specialists using digital monoscopic fundus images.

Frequency doubling technology perimetry (FDT; Humphrey Matrix; Carl Zeiss Meditec Inc, Dublin, CA, USA) testing using the N-30-1 screening program was performed when a participant had an elevated IOP ≥ 22 mmHg or a glaucomatous optic disc with typical loss of the neuroretinal rim by slit-lamp biomicroscopy or fundus photography (cup-to-disc ratio >0.7; inter-eye cup asymmetry >0.2; or neuroretinal rim notching, focal thinning, disc hemorrhaging, or vertical elongation of the optic cup). FDT testing was repeated once if initial testing was deemed unreliable. The presence of an FDT examination abnormality was determined by comparison with the manufacturer’s internal normative database. Test results for visual field (VF) locations were classified into probability levels based on age-corrected normative values and depicted on a colored gray scale. An abnormal examination was defined by the presence of at least one test point of reduced sensitivity with *P* < 1%, *P* < 0.05, or “not seen at maximum” on the total deviation plot. 

Cataract was defined as the presence of nuclear, cortical, or posterior subcapsular opacity at least in one eye with a best-corrected visual acuity of <0.8 [7]. Glaucoma was diagnosed using the criteria of International Society of Geographical and Epidemiological Ophthalmology (ISGEO) [9]. Diabetic retinopathy was defined as the presence of one or more retinal microaneurysms or retinal blot hemorrhages with or without more severe lesions (hard exudates, soft exudates, intraretinal microvascular abnormalities, venous bleeding, new retinal vessels, or fibroproliferations) [10]. A patient was defined as having early AMD when a fundus photograph met either or both of the following criteria: (1) the presence of soft indistinct drusen or reticular drusen, or (2) the presence of hard or soft distinct drusen with pigmentary abnormalities (increased pigmentation or hypopigmentation of retinal pigment epithelium) in the absence of signs of late AMD. Late AMD included the presence of signs of wet AMD or geographic atrophy, and wet AMD was defined as retinal pigment epithelial detachment or serous detachment of the sensory retina, subretinal, or sub-RPE hemorrhage, and subretinal fibrous scarring. Geographic atrophy was defined as a circular discrete area (at least 175 microns in diameter) of retinal depigmentation with visible choroidal vessels in the absence of signs of wet AMD. The specific diagnostic criteria adopted for this survey has been previously described [7].

### 2.3. Otolaryngology Examinations and Audiometric Tests

Ear examinations were performed using a 4-mm 0-angled rigid endoscope (Xion GmbH, Berlin, Germany) and the ML 150 vision system (JRMed Trade Co., Seoul, Korea) by trained otolaryngologists and otolaryngology residents. Examination data were periodically evaluated by a quality control committee. Air-condition pure-tone thresholds were measured in a double-walled soundproof booth (CD-600; Sontek, Paju, Korea) using an automatic audiometer (GSI SA-203; Entomed Diagnostics AB, Lena Nodin, Sweden) for each ear at six frequencies (500, 1000, 2000, 3000, 4000, and 6000 Hz) by well-trained examiners. Hearing loss was defined as pure-tone average thresholds at 500, 1000, 2000, 3000, 4000, and 6000 Hz averaged for both ears of > 40-dB. Hearing impairment was categorized by frequency (low or high) and severity (mild or moderate-to-profound). Low-frequency pure-tone was defined as the average pure-tone threshold at 500, 1000, and 2000 Hz. High-frequency pure-tone was defined as the average pure-tone threshold at 3000, 4000, and 6000 Hz, and mild hearing impairment was defined as an unaided pure-tone mean of 26–40 dB. Moderate-to-profound hearing impairment was defined as an unaided pure-tone mean of >40 dB.

A history of noise exposure was obtained using questionnaire responses. Occupational exposure to noise was as a history of loud noise at work for >3 months that required speaking with a loud voice. Environmental exposure to noise was defined as exposure to loud noise for >5 hour/week outside work requiring speaking with a loud voice.

### 2.4. Measurements

Those with a smoking history were categorized as non-smokers, ex-smokers, or current smokers. Alcohol consumption was determined by asking participants about drinking behavior during the previous year. Height, weight, and waist circumference were measured during a physical examination. Body mass index was calculated by dividing subject weight in kilograms by the square of subject height in meters. Systolic and diastolic blood pressures were measured in the sitting position after a minimum of 5 minutes of rest. Fasting serum glucose, triglyceride (TG), total cholesterol, and high-density lipoprotein cholesterol (HDL-C) levels were measured using conventional methods. 

### 2.5. Statistical Analysis

The statistical analysis was performed using IBM SPSS Statistics for Windows/Macintosh, Version 23.0 (IBM Corp., Armonk, NY, USA). *P*-values of < 0.05 were considered significant. The data analysis was performed using weighted data; SEs of mean population estimates were calculated using Taylor linearization methods. Participant characteristics were expressed as means and SEs for continuous variables and as frequencies and percentages for categorical variables.

Group baseline demographic information and clinical parameters were compared using Pearson’s chi-square test for categorical variables and the general linear model for continuous variables. Univariate and multivariate logistic regression analyses were used to assess the risk ratios of specific ocular diseases according to the presence or absence of hearing impairment. Logistic regression analysis was fitted with increasing degrees of adjustment using a model adjusted for age and sex (Model 1) and a model adjusted for age, sex, alcohol, smoking, systolic BP, diastolic BP, waist circumference, BMI, fasting blood sugar, total cholesterol, HDL, and TG (Model 2). Risk factors for concurrent specific ocular disease and hearing loss were also analyzed using univariate and multivariate logistic regression analyses.

## 3. Results

Of the 25,534 patients, 17,957 participants were eligible for both full ocular examinations and ENT examinations. The analysis excluded 2111 people with a history of occupational exposure to noise and 360 people with a history of environmental exposure to noise. In addition, 74 people with external auditory canal stenosis, 428 with perforation of the tympanic membrane, 348 with cholesteatoma, and 113 with chronic otitis media were excluded. We also excluded 1220 participants who were unable to undergo eardrum examination and 1236 who had no audiometric test. Finally, 12,899 people were eligible for inclusion in the study.

Table 1 summarizes the baseline characteristics of the study participants. The proportion of men was significantly higher in the hearing-impaired group, and mean age was higher and mean income and educational levels were lower in the hearing-impaired group than in the normal hearing group (all, *P* < 0.05).

Table 2 shows the ocular characteristics of study participants. Intraocular pressure was lower, refractive error was more hyperopic, VCDR were greater, non-corrected visual acuity was lower, and proportions with a cataract or a visual field abnormality were larger in the hearing-impaired group (all, *P* < 0.05).

Table 3 shows the weighted prevalences of cataract, glaucoma, DR, early AMD, and late AMD in the normal hearing and hearing-impaired groups. The weighted prevalences of cataract and glaucoma in the hearing-impaired group were 25.0% and 7.6%, respectively, which was significantly higher than prevalences in the normal hearing group (3.6% and 3.2%, respectively, both *P* values < 0.001). The weighted prevalences of diabetic retinopathy were similar in the two groups (*P* = 0.740), and the weighted prevalences of early AMD and late AMD were significantly higher in the hearing-impaired group (10.6% and 1.6%, respectively) than in the normal hearing group (2.8% and 0.2%, respectively; both *P* values < 0.001).

The effects of hearing impairment on the weighted ORs of specific ocular diseases are shown in Table 4. In models not adjusted for any demographic or clinical variables, hearing loss significantly increased the risk of cataract, glaucoma, and early and late AMD (all, *P* < 0.001). However, in Model 1, which was adjusted for age and gender, hearing loss only increased the risk of cataract 1.373-fold (95% CI, 1.118–1.678, *P* = 0.003), and in Model 2, which was adjusted for age, sex, alcohol, smoking, systolic BP, diastolic BP, waist circumference, BMI, fasting blood sugar, total cholesterol, HDL, and triglyceride, hearing loss increased the risk of cataract 1.408-fold (95% CI 1.126–1.762, *P* = 0.003).

In Model 1, hearing impairment in the low frequency region increased the risk of cataract 1.377-fold (95% CI, 1.119–1.693, *P* = 0.002), and high frequency hearing impairment increased the risk of cataract 1.348-fold (95% CI, 1.114–1.630, *P* = 0.002). Mild hearing impairment increased cataract risk 1.420-fold (95% CI, 1.147–1.757, *P* = 0.001), and moderate-profound hearing impairment increased cataract risk 1.714-fold (95% CI, 1.350–2.178, *P* < 0.001) as compared with the normal hearing group (Table 5).

The risk factors associated with the simultaneous presentation of a specific eye disease and hearing impairment and their odds ratios are presented in Table 6. Age was the only risk factor of concurrent cataract and hearing impairment, and showed a 6.574-fold (95% PI, 5.254–8.226) increased risk per decade. Glaucoma and hearing impairment was associated with age, male sex, and triglyceride, with a 6.574-fold increase in risk per decade, a 3.144-fold increase in risk for men, and a 1.003-fold increase in risk per 1 mg/dL of triglyceride increase. The risk of concurrent diabetic retinopathy and hearing impairment was associated with age, ex-smoker, systolic BP, and fasting blood sugar (ORs, 3.033, 3.144, 1.284, and 1.021, respectively). A high BMI was associated with a 0.781-fold reduced risk of concurrent diabetic retinopathy and hearing impairment. Age and triglyceride were associated with increased risk of concurrent early AMD and hearing impairment (3.693-fold/decade and 1.002-fold per 1 mg/dL, respectively). Age and systolic BP were associated with increased risk of late AMD and hearing impairment (3.727-fold per decade and 1.524-fold per 10 mmHg, respectively).

## 4. Discussion

Ocular diseases that are severe enough to cause visual impairment may severely reduce quality of life. Hearing loss also impair the quality of life of the elderly, and the problem is even greater if the dual senses are lost. Thus, the identification of common risk factors of these multiple disorders might lead to the developments of effective preventive interventions [11,12]. We investigated the relationship between sight loss and hearing loss using KNHANES V data and sought to identify the risk factors of such comorbidities.

### 4.1. Cataract and Hearing Impairment

Various opacities of cortical, nuclear, and subcapsular lesions were more common in the hearing-impaired group than in the normal group. In addition, covariate-corrected models showed hearing impairment was associated with an elevated risk of cataracts. Klein et al. reported risk factors for concurrent age-related cataract and hearing loss by analyzing data obtained during the large population-based Beaver Dam Eye Study [11]. Any type of cataract in combination with hearing loss in either ear was observed in 27.8% of the overall population and this increased consistently with age. In addition, by using multivariable models, they found smoking, heavy drinking, and other risk factors were associated with cataract and hearing loss. The authors suggested that modification of certain lifestyle habits might reduce risk.

In the elderly, hearing loss usually involves high-frequency hearing loss, which means that our analysis of the relation between high and low frequency hearing impairment and cataracts is important. Degeneration of the cochlear lateral wall, and particularly of the stria vascularis, is considered a major cause of presbycusis, which is sometimes referred to as “metabolic presbycusis”[13]. A syndrome of cochleosaccular degeneration of the inner ear and progressive cataracts inherited as an autosomal dominant trait has been described in the literature [14]. However, more studies are needed to determine the systemic status and risk factors of concurrent cataracts and hearing loss in older adults.

In Models 1 and 2, mild hearing impairment increased the risks of cataracts by 1.420 and 1.328-fold and moderate-profound hearing impairment increased risks by 1.714 and 1.617-fold, respectively, which means the greater the severity of hearing impairment, the greater the risk of cataracts. Since the majority of cataracts are surgically removable, we suggest patients with severe hearing impairment be checked for the presence of cataract.

### 4.2. Glaucoma and Hearing Impairment

Kremmer et al. reported that patients with normal-tension glaucoma showed a significantly higher prevalence of hearing loss [15]. Others have reported an association between pseudoexfoliation glaucoma and hearing impairment [16,17,18,19,20,21]. However, the relationship between glaucoma and hearing loss is controversial. A recent study have reported that patients with pseudoexfoliation syndrome/pseudoexfoliation glaucoma or patients with POAG did not show a significant difference in hearing compared to controls after considering possible confounders [22]. In the present study, visual field impairment confirmed by FDT was more frequent in hearing-impaired group compared to normal hearing group. And the results obtained showed the prevalence of primary open angle glaucoma was higher in the hearing-impaired group, but our covariate-adjusted models showed hearing impairment did not significantly increase the risk of glaucoma.

The risk factors associated with concurrent glaucoma and hearing loss in the present study were age, male sex, and TG level. Thus, when a patient in the early stages of glaucoma with hearing impairment has a high TG level, treatment for hyperlipidemia might helpfully reduce the progressions of both diseases. Further research into the pathophysiology of glaucoma and hearing loss comorbidity is required undertaken.

### 4.3. DR and Hearing Impairment

In diabetes mellitus, generalized vascular pathology is believed to cause changes in the inner ear [23], and a report on a deaf diabetic patient with confirmed microangiopathy seems to support this presumption [24]. In addition, it was also suggested that diabetic sensorineural hearing loss results from microangiopathic involvements of the endolymphatic sac and/or basilar membrane vessels based on the histopathologic findings in temporal bones of eight diabetics and ten normal controls [25]. The relationship between diabetic retinopathy and hearing loss has been reported in several studies [26,27,28]. However, reports often conflict. Studies on diabetes and hearing loss often conclude that an association exists, but many researchers have found results difficult to interpret, for example, the differentiation of diabetes-related hearing loss and normal aging is not straightforward and our understanding of whether hearing deterioration depends on diabetes severity or metabolic control is limited [26].

In the present study, the prevalence of diabetic retinopathy was similar in the hearing-impaired and normal hearing groups, indicating hearing loss is not associated with an increased risk of diabetic retinopathy. Risk factors associated with combined hearing loss and DR were age, ex-smoking, high systolic blood pressure, low BMI, and high fasting blood sugar. Monitoring auditory function and ophthalmic examination might be beneficial for the management of diabetic patients, especially those with these risk factors.

### 4.4. AMD and Hearing Impairment

In a previous large study that uses data from the Beaver Dam Offspring Study, early AMD was found to be significantly associated with hearing impairment [29], which is in-line with the increased risk of late AMD observed among individuals with hearing loss in the Beaver Dam Eye Study [30]. In the present study, multivariate analysis showed the presence of hearing impairment did not increase the risk of early or late AMD. Nevertheless, early AMD with concurrent hearing impairment was associated with an increase in triglyceride levels, and late AMD with concurrent hearing impairment was associated with an increase in systolic BP. In a previous study that used fluorescein angiography, it was mentioned that the impaired circulation modulates the association between AMD with hearing impairment [31], and in another study melanin dysfunction was suggested to underlie the pathologies of both diseases [32]. More specifically, the authors suggested melanin dysfunction might lead to the apoptosis of retinal pigment epithelial cells in AMD [33,34], and that ageing of the cochlear, which contains large amounts of melanin, might be responsible for age-related hearing deterioration [35,36].

### 4.5. Limitations

The present study has several limitations that warrant mention. First, it is inherently limited by its cross-sectional design that limits the ability to explore the associations between hearing loss and ocular diseases. Second, although patients with chronic otitis media, ruptured tympanic membrane, and cholesteatoma were excluded, conductive hearing loss and sensorineural hearing loss could not be completely differentiated, and thus, we cannot affirm that all patients with conductive hearing loss were excluded. Third, unmeasured or residual confounding factors may have caused unexpected analytical bias. Fourth, VF was examined by FDT rather than by Humphrey field analysis, which is the test of choice for VF testing. However, FDT is a fast, reliable, and large-scale screening method that can detect glaucomatous VF defects earlier than standard automated perimetry [37]. Fifth, angle status was assessed using Van Herick’s method and not by gonioscopic examination. On the other hand, the strengths of our study are that it is representative of the Korean population, has a relatively large sample size, and a high participation rate.

## 5. Conclusions

Hearing loss and ocular diseases often co-occur, but the multivariate models used in the present study showed hearing loss was only associated with an increased risk of cataracts. The factors affecting the coexistence of cataract, glaucoma, diabetic retinopathy, or AMD and hearing loss differed by ocular disease, except for age. Previous studies have shown individuals with visual and auditory problems are at greater risks of physical, mental, and functional morbidities. We recommend auditory screening be performed on patients with impaired vision, and that hearing-impaired patients be checked for vision problems. In addition, we suggest that the common risk factors presented in this study be considered when screening limits are determined.

## Figures and Tables

**Table 1:** Characteristics of the study population.

		No Hearing Impairment	Hearing Impairment	*P*
Weighted prevalence		91.0% (0.3%)	9.0% (0.3%)	
Demographic characteristics	Age	42.18 (0.217)	66.02 (0.542)	<0.001
	Sex (Male:Female)	45.2%:54.8%	55.6%:44.4%	<0.001
	Annual income			
	Lowest quartile	12.1%	40.6%	<0.001
	Second quartile	27.1%	27.2%	
	Third quartile	30.5%	17.8%	
	Highest quartile	30.3%	14.4%	
	Education			
	High school or lower	21.2%	68.4%	<0.001
	High school graduate or higher	40.9%	20.4%	
	College graduate or higher	37.9%	11.2%	
	Occupation			
	Office workers *	31.7%	16.4%	<0.001
	Others ^†^	60.5%	72.3%	
	Unemployed	7.8%	11.3%	
Data obtained from the questionnaire	Smoking			
	Current smoker	23.5%	22.5%	<0.001
	Ex-smoker	16.0%	31.3%	
	Non-smoker	47.8%	43.4%	
	Alcohol			
	AUDIT score	6.83 (0.091)	6.54 (0.318)	0.367
	No:Yes	15.5%:84.1%	20.1%:77.7%	<0.001
Anthropometrics	BMI, kg/m^2^	23.67 (0.051)	23.68 (0.099)	0.988
	Waist circumference, cm	80.53 (0.154)	83.51 (0.321)	<0.001
	Systolic BP, mmHg	116.43 (0.228)	127.63 (0.575)	<0.001
	Diastolic BP, mmHg	76.20 (0.169)	75.95 (0.331)	0.486
Metabolites, mg/dL	Fasting serum glucose	95.60 (0.258)	103.19 (0.770)	<0.001
	Triglyceride	129.79 (1.462)	148.28 (4.937)	<0.001
	Total cholesterol	187.08 (0.486)	192.24 (1.285)	<0.001
	HDL cholesterol	49.55 (0.149)	47.20 (0.404)	<0.001

Weighted mean (standard errors). Abbreviations: AUDIT, alcohol use disorders identification test; BMI, body mass index; BP, blood pressure; HDL, high-density lipoprotein. * 1) Management occupations 2) professional and related occupations, and 3) office occupations ^†^ 1) Service occupations, 2) sales occupations, 3) agriculture and fishery, 4) construction, extraction, maintenance, and repair occupations, and 5) production, transportation, and material moving occupations. Generalized linear model and Chi-square test.

**Table 2:** Ocular parameters, right eyes.

		No Hearing Impairment	Hearing Impairment	*P*
Intraocular pressure, mmHg		13.94 (0.062)	13.70 (0.106)	0.015
Spherical equivalent, diopters		−1.40 (0.314)	0.04 (0.060)	<0.001
Vertical Cup-to-disc ratio		0.368 (0.0025)	0.392 (0.0051)	<0.001
Non-corrected visual acuity	≥0.8	75.0%	53.5%	<0.001
	≥0.32	18.9%	35.9%	
	<0.32	4.5%	8.6%	
Lens opacity	Cortical opacity, %	3.4%	10.6%	<0.001
	Nuclear opacity	10.4%	30.8%	
	Anterior (sub)capsular opacity	0.3%	1.1%	
	Posterior subcapsular opacity	0.1%	0.5%	
	Mixed Opacity	1.9%	9.9%	
FDT N-30	Number of *P* < 0.5% points	0.39 (0.044)	1.02 (0.118)	<0.001
	Number of abnormal points *	0.62 (0.055)	1.68 (0.155)	<0.001

Weighted mean (standard errors). Abbreviations: VCDR, vertical cup-to-disc ratio; IOP, intraocular pressure; FDT, frequency doubling technology perimetry. * Number of abnormal points: < 0.5%, < 1%, not seen at maximum. Generalized linear model and Chi-square test.

**Table 3:** Weighted prevalences of eye diseases in those without and with hearing impairment.

	No Hearing Impairment	Hearing Impairment
Cataract		
No	96.4%	75.0%
Yes	3.6%	25.0%
*P*	<0.001	
Glaucoma		
No	96.8%	92.4%
Yes	3.2%	7.6%
*P*	<0.001	
Diabetic retinopathy		
No	82.5%	81.5%
Yes	17.5%	18.5%
*P*	0.740	
Early AMD		
No	97.2%	89.4%
Yes	2.8%	10.6%
*P*	<0.001	
Late AMD		
No	99.8%	98.4%
Yes	0.2%	1.6%
*P*	<0.001	

Chi-square test.

**Table 4:** Weighted ORs of eye diseases according to the presence of hearing impairment.

	Odds Ratio (95% Confidence Interval, *P*)		
	Crude	Model 1	Model 2
Cataract	10.073 (8.498–11.941, <0.001)	1.373 (1.118–1.687, 0.003)	1.408 (1.126–1.762, 0.003)
Glaucoma	2.929 (2.270–3.778, <0.001)	1.104 (0.825–1.476, 0.505)	1.043 (0.767–1.417, 0.790)
Diabetic retinopathy	1.150 (0.715–1.850, 0.564)	0.721 (0.425–1.223, 0.224)	0.762 (0.448–1.295, 0.315)
Early AMD	4.704 (3.677–6.017, <0.001)	0.898 (0.666–1.211, 0.480)	0.946 (0.686–1.304, 0.734)
Late AMD	6.400 (3.084–13.282, <0.001)	1.106 (0.448–2.729, 0.827)	1.058 (0.421–2.661, 0.904)

Abbreviations: OR, odds ratio; CI, confidence interval. ORs (95% CIs, *P*-value). Model 1 adjusted by age and sex. Model 2 adjusted by age, sex, alcohol, smoking, systolic BP, diastolic BP, waist circumference, BMI, fasting blood sugar, total cholesterol, HDL, and triglyceride. Logistic regression analyses.

**Table 5:** Weighted ORs for cataract according to impaired frequency and the severity of hearing impairment.

			Odds Ratio (95% Confidence Interval, *P*)	
		Crude	Model 1	Model 2
Frequency	Low frequency	8.319 (7.006–9.879, <0.001)	1.377 (1.119–1.693, 0.002)	1.345 (1.064–1.701, 0.013)
	High frequency	9.044 (7.772–10.525, <0.001)	1.348 (1.114–1.630, 0.002)	1.315 (1.074–1.610, 0.008)
Severity	Normal	1.000	1.000	1.000
	Mild impairment	8.044 (6.636–9.750, <0.001)	1.420 (1.147–1.757, <0.001)	1.328 (1.058–1.667, <0.001)
	Moderate-profound impairment	17.250 (14.340–20.752, <0.001)	1.714 (1.350–2.178, <0.001)	1.617 (1.256–2.083, 0.014)
	*P* for trend	<0.001	<0.001	<0.001

Abbreviations: OR, odds ratio; CI, confidence interval. ORs (95% CIs, *P*-value). Normal, mean hearing threshold ≤26 dB; mild impairment, 26 < mean hearing threshold ≤ 40 dB; moderate-profound impairment, mean hearing threshold >40 dB. Model 1 adjusted by age and sex. Model 2 adjusted by age, sex, alcohol, smoking, systolic BP, diastolic BP, waist circumference, BMI, fasting blood sugar, total cholesterol, HDL, and triglyceride. Logistic regression analyses.

**Table 6:** Risk factors for concurrent eye diseases and hearing impairment.

	Risk Factors	Odds Ratio (95% Confidence Interval)	*P*
Cataract + hearing impairment	Age, per 10 years	6.574 (5.254–8.226)	<0.001
Glaucoma + hearing impairment	Age, per 10 years	3.570 (2.857–4.461)	<0.001
	Male gender (versus female gender)	3.144 (1.119–8.829)	0.030
	Triglyceride, per 1 mg/dL	1.003 (1.001–1.005)	0.001
Diabetic retinopathy + hearing impairment	Age, per 10 years	3.033 (2.229–4.127)	<0.001
	Ex-smoker (versus never)	3.144 (1.203–8.218)	0.020
	Systolic BP, per 10mmHg	1.284 (1.042–1.581)	0.019
	BMI, per 1kg/m^2^	0.781 (0.621–0.982)	0.034
	Fasting blood sugar, per 1 mg/dL	1.021 (1.016–1.027)	<0.001
Early AMD + hearing impairment	Age, per 10 years	3.693 (3.098–4.403)	<0.001
	Triglyceride, per 1 mg/dL	1.002 (1.001–1.003)	0.003
Late AMD + hearing impairment	Age, per 10 years	3.727 (1.909–7.278)	<0.001
	Systolic BP, per 10mmHg	1.524 (1.091–2.129)	0.014

Abbreviations: BMI, body mass index; BP, blood pressure; HDL, high-density lipoprotein. Model adjusted by age, sex, alcohol, smoking, systolic BP, diastolic BP, waist circumference, BMI, fasting blood sugar, total cholesterol, HDL, and triglyceride. Logistic regression analyses.
